# Permanent Complications After Dermal Fillers: Risks, Prevention, and Management Strategies

**DOI:** 10.1007/s00266-026-05989-8

**Published:** 2026-06-15

**Authors:** Simran K. Chandawarkar, Ibrahim Amjad

**Affiliations:** 1 Northeast Ohio Medical University, 4109 OH-44, Rootstown, OH 44272 USA; 2 AmjadPlastics, Miami, FL USA

**Keywords:** Dermal fillers, Adverse effects, Permanent side effects, Complications, Minimally invasive, Esthetic surgery

## Abstract

**Background:**

Dermal fillers are widely utilized in esthetic medicine for soft tissue augmentation and facial rejuvenation. Generally safe, complications are mostly minor. Permanent complications, though rare, are disastrous and overwhelmingly caused by insufficient understanding of facial anatomy, wrong filler selection, technique, and poor diagnosis and management. This review aims to bridge the knowledge gaps that lead to permanent complications, provide guidelines to avoid them, and create best practices to manage them should they occur.

**Methods:**

A PRISMA-guided systematic search of PubMed (2000–2024) was conducted using relevant search terms of English-language publications reporting irreversible outcomes or complications of fillers persisting beyond 12 months.

**Results:**

Blindness, stroke, chronic granulomatous inflammation, and irreversible tissue necrosis represent the most severe outcomes. The preponderance of evidence indicates that anatomic injection site, wrong technique, filler permanence, rather than injected volume alone, are the principal determinants of irreversible injury. HA fillers remain preferable due to reversibility; permanent fillers pose increased long-term complication risk. The following synthesis examines anatomically based injection safety principles, product selection criteria by location, and complication prevention strategies supported by peer-reviewed literature.

**Conclusions:**

Safe and effective dermal filler practice is grounded in anatomic precision, meticulous technique, informed product selection, and preparedness to manage complications. Continuous education, adherence to evidence-based protocols, and awareness of high-risk anatomic regions could significantly reduce adverse outcomes.

**Level of Evidence IV:**

This journal requires that authors assign a level of evidence to each article. For a full description of these Evidence-Based Medicine ratings, please refer to the Table of Contents or the online Instructions to Authors www.springer.com/00266.

**Supplementary Information:**

The online version contains supplementary material available at https://doi.org/10.1007/s00266-026-05989-8.

## Introduction

Permanent adverse effects of dermal fillers (DFs) result in disastrous and devastating consequences for the patient. The percentage incidence rates are low, but with the astronomical rise in the DFs used globally, the actual number of events would be expected to be alarmingly high. In 2024, approximately 6,264,287 dermal filler procedures were performed, and in 2023, they accounted for $5B in revenue and are estimated to be $14B by 2032 [1]. DFs have now become the second most performed minimally invasive cosmetic procedure in the USA [1]. Provider profiles offering DFs have changed, raising concerns that underqualified providers, non-physicians, and even med spa operators offering DFs could put patients at risk [2]. The increased global demand, absence of regulation, and faulty practices, compounded by cheap and untested substitutes, increase the risks of all complications, including the permanent ones, significantly. Left unrecognized or undertreated, serious adverse effects could result in catastrophic deficits [3]. Several published reports focus on different side effects of fillers, either individually or related to their biochemical dynamics. Moreover, a recent FDA panel in August 2025 released that they require more data on the use of fillers in certain areas of the body, further underscoring the caution we need to have when using these. This work provides both new injectors and patients with a comprehensive review of the etiology of permanent adverse effects, their management, and prevention strategies. Based on these results, we describe a Clinical Decision Tree to facilitate safe practices and help injectors avoid unwanted effects. It explains anatomic considerations, filler selection, proper injection techniques, and warning signs of complications for timely diagnosis and targeted intervention. These measures could help both new and established providers prevent complications in general, educate patients, and limit damage from serious complications should they occur.

## Methods

A PRISMA-guided systematic search of PubMed (2000–2024) was conducted. Inclusion criteria were English-language studies reporting irreversible outcomes or complications persisting beyond 12 months. Case reports, series, reviews, and meta-analyses were included. Specifically, searches used keywords: “filler injection” or “dermal filler” AND “permanent adverse effects,” “permanent side effects,” “long-term effects,” “complications,” “costs of treatment of adverse effects,” and “prevention” and the relevant studies were reviewed. An Internet search was performed to uncover some of the common misperceptions that existed about DF in general.

## Results

**Fig. 1:**
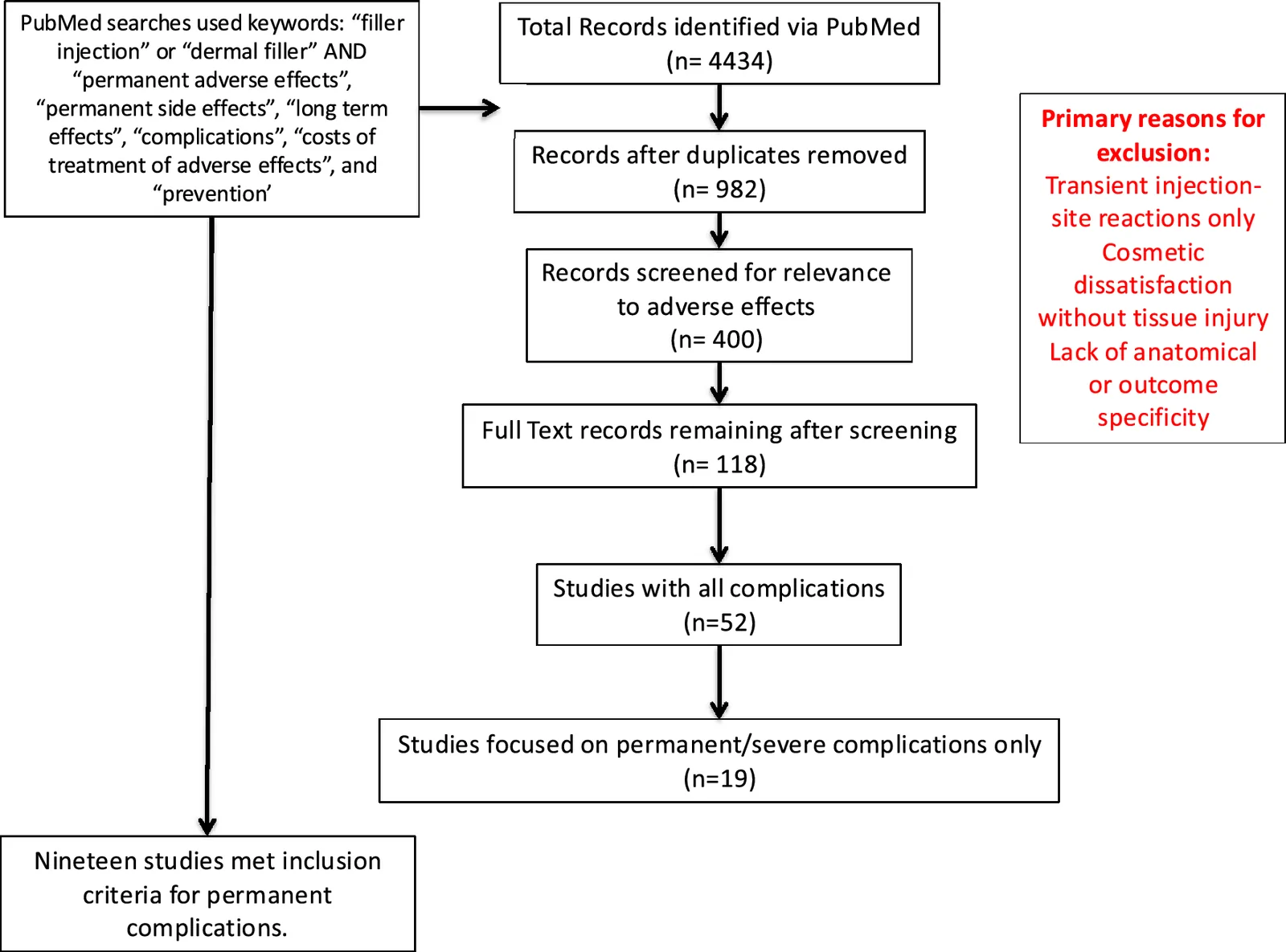
PRISMA guidelines applied for the study

A total of 4434 articles were screened, and after accounting for relevance and duplicates, and subsequent screening (Fig. 1), 19 full-text articles that pertained to permanent/serious adverse effects of dermal fillers were analyzed.

**Fig. 2:**
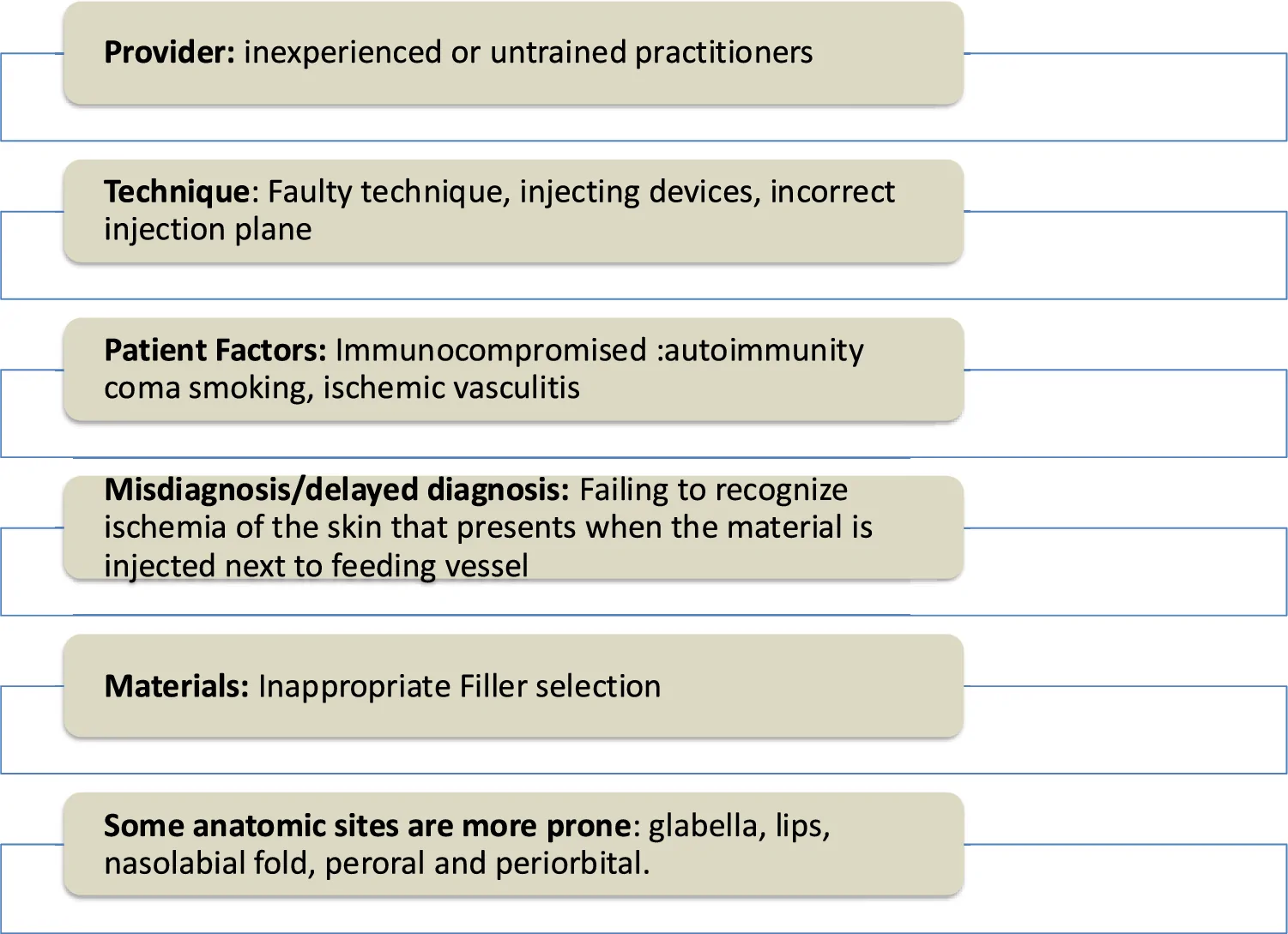
Risk factors for permanent side effects

**Fig. 3:**
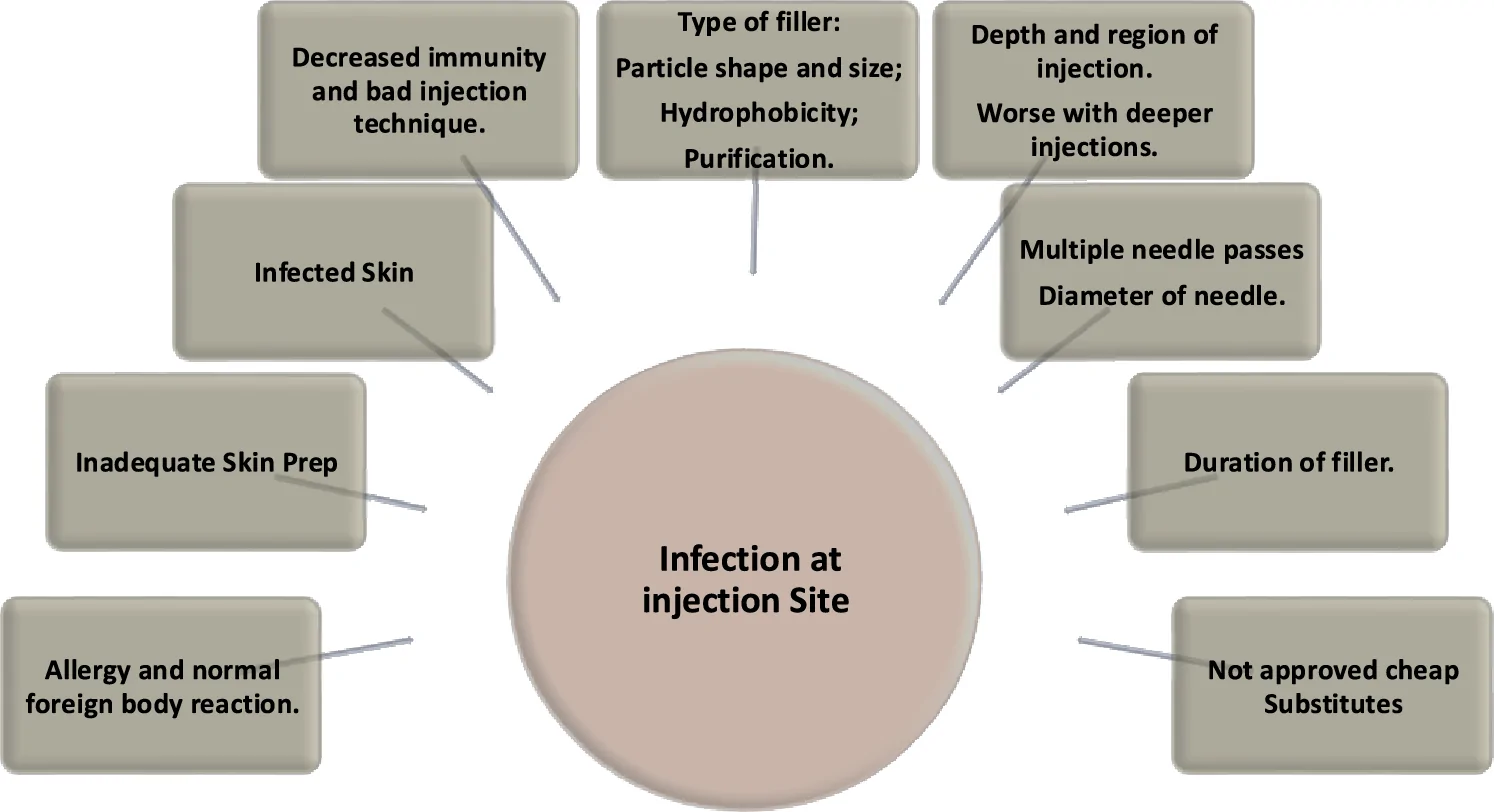
Conditions that aggravate biofilm infection after soft tissue filler injection.

**Fig. 4:**
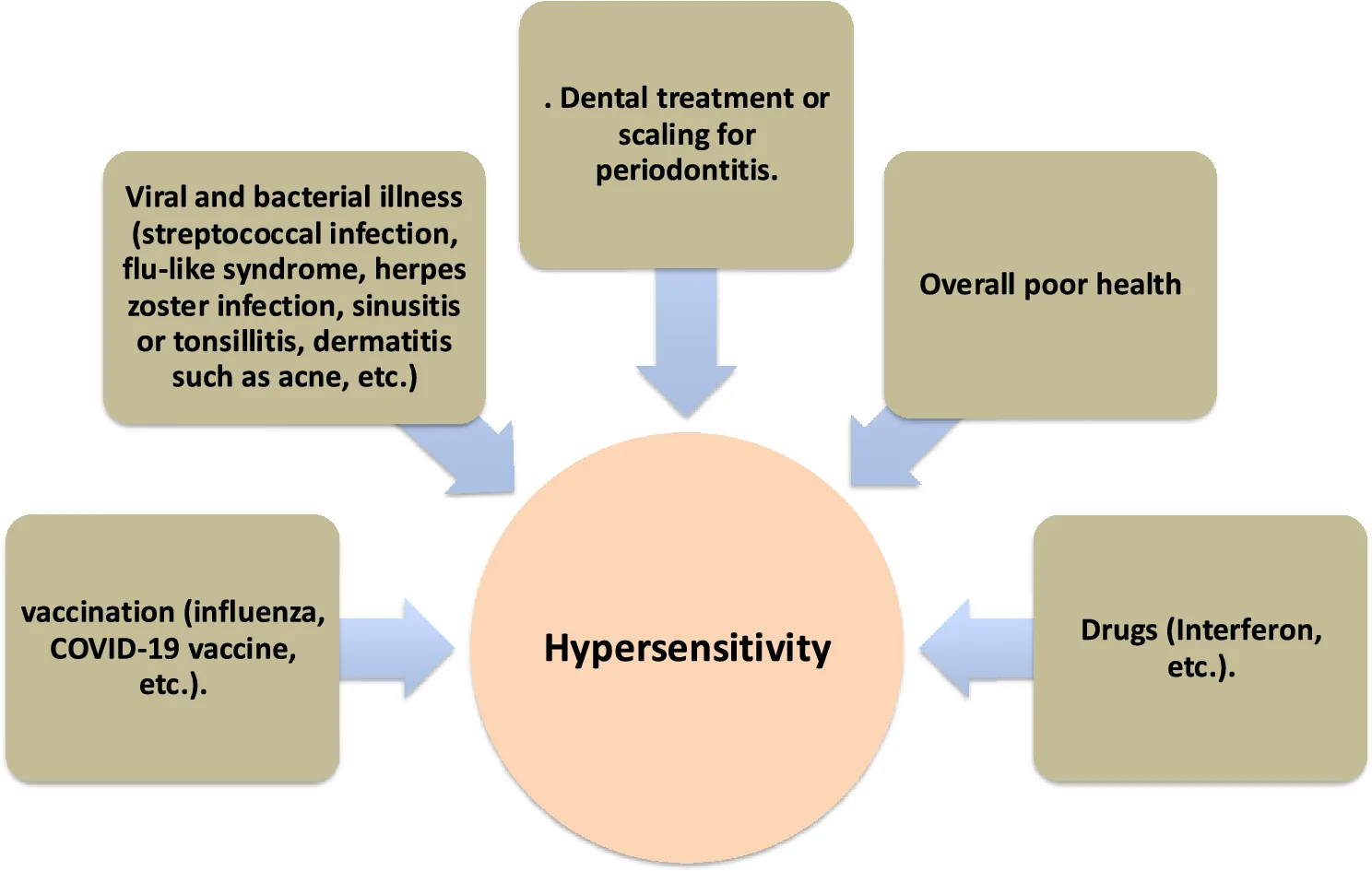
Immunological triggers that may cause hypersensitivity after filler injection

**Fig. 5:**
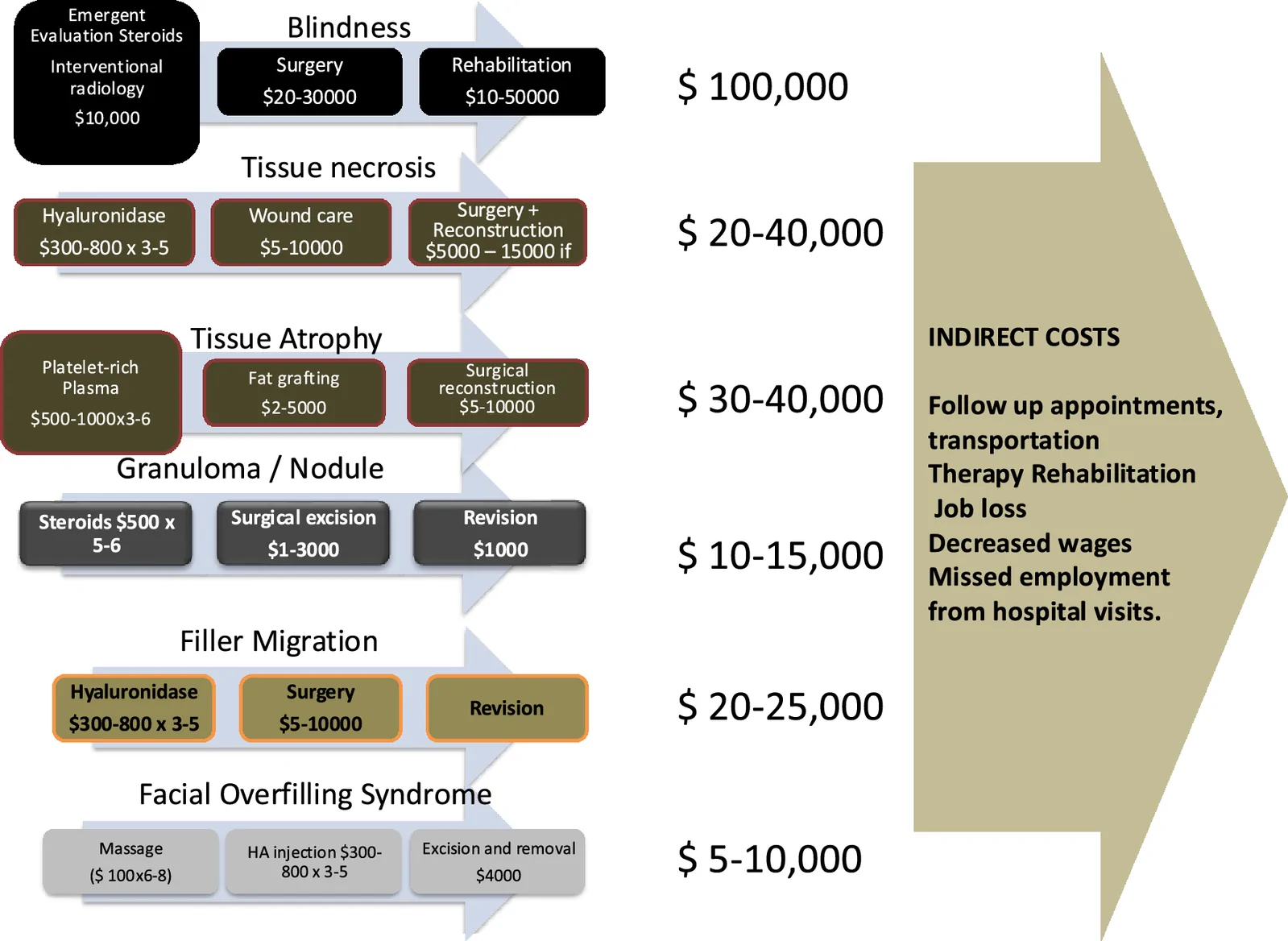
Treatment costs of severe adverse effects of dermal fillers

**Fig. 6:**
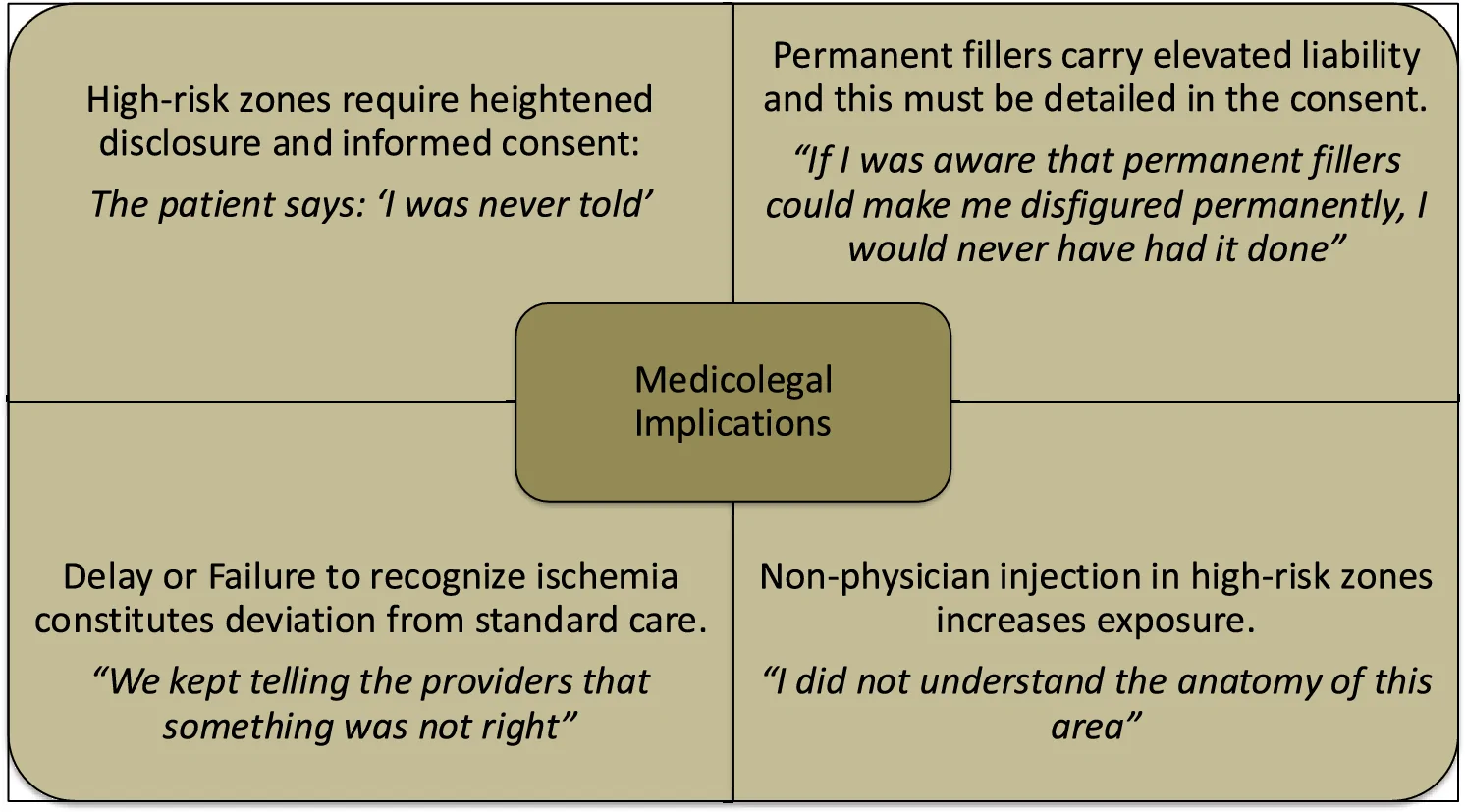
Common reasons that lead to a lawsuit following DF-related complications

Incidence of permanent complications from injection of dermal fillers is low (Table 1); most authors emphasize that the true incidence is likely underestimated due to voluntary reporting and medicolegal disincentives. The permanent adverse effects identified in Table 2 are largely due to vascular compromise, foreign body-like occlusion or pressure, or damage to tissues or even nerves. Certain anatomic sites were at higher risk: Injections of DF in the glabella and nasal dorsum were most frequently implicated in blindness; the nasolabial fold and forehead were associated with cerebral embolic events (Table 3). Of all filler materials, permanent fillers (PMMA, silicone) were disproportionately associated with chronic inflammatory, vascular, and deforming complications (Table 4). Major risk factors included provider experience, injection technique, patient-specific factors, filler type, and certain anatomic areas more prone to complications (Fig. 2). Key findings are consistently reported across systematic reviews and meta-analyses included: Blindness is the most devastating and consistently irreversible complication; permanent fillers disproportionately cause chronic complications; anatomic location outweighs volume injected in risk prediction; delayed recognition of vascular compromise worsens permanence; HA fillers dominate case reports due to usage frequency, not intrinsic danger. In addition to the main permanent complications, two others, hypersensitivity and infection at the injection site, were completely preventable by meticulous history-taking, proper injection technique, and early diagnosis (Figs. 3 and 4). Studies revealed that the costs of treating severe complications were substantially high (Fig. 5), even without calculating indirect costs of lost wages and time off. Medicolegal implications mainly included disclosure and informed consent, permanent fillers, misdiagnosis, and provider training (Fig. 6). Common myths about DFs discussed in the literature included: “*All DFs are safe.”* Since the occurrence of severe complications is rarely reported (unless patients need hospital admission), the perception of safety is high. Currently, even severe complications are not required to be reported, with very few published reports in the literature [4]. “*All filler-related complications are self-limiting”*: Minor complications do resolve, but serious vascular or tissue injury can produce lifelong adverse effects. “*Injection of fillers is so easy, anyone can do it.”* Though relatively easier than surgery, injection skill and proper filler selection are critical factors to prevent complications. “*All DF-related complications are easy to treat, even severe ones.”* Untrue, especially for severe adverse effects that, if undertreated or misdiagnosed, could result in permanent deficits.

**Table 1:** Incidence and risk trends (where quantified)

Finding	Evidence summary
Overall complication rate	Estimated 0.1–1% for significant complications; mild events common but underreported
Vascular occlusion	Rare (<0.05%) but disproportionately represented in severe outcomes
Blindness	Estimated <1 per 10,000 injections; typically irreversible
Permanent fillers	Significantly higher rate of late and permanent complications than HA fillers

**Table 2:** Permanent complications of dermal fillers

Complication	Pathophysiology	Permanence	Key evidence
Blindness	Retrograde embolization to ophthalmic/retinal artery	Nearly always permanent	Tran et al.; Sito et al.
Stroke	Cerebral arterial embolism	Permanent	Sito et al.
Skin necrosis with scarring	Arterial occlusion → ischemia	Permanent if delayed	Hong et al.
Chronic granuloma	Foreign-body reaction (PMMA, silicone, PLLA)	Permanent	Kunjur & Witherow
Filler migration	Poor encapsulation/permanent material	Permanent	Haneke
Ophthalmoplegia	Ischemic cranial nerve injury	Usually permanent	Zein et al.
Facial deformity	Chronic inflammation/fibrosis	Permanent	Kunjur & Witherow

**Table 3:** Anatomic sites prone to complications of dermal fillers

Anatomical site	Permanent complication	Risk level
Glabella	Blindness, necrosis	Highest
Nose, nasal tip	Blindness, scarring	Very high
Forehead	Blindness	Very high
Nasolabial fold	Stroke, necrosis	High
Tear trough	Ophthalmoplegia, vision loss	High
Lips	Rare necrosis	Moderate

**Table 4:** Filler type prone to permanent complications

Filler Type	Reversibility	Permanent complication risk
Hyaluronic acid	Yes	Low (but blindness irreversible)
CaHA	Partial	Moderate
PLLA	No	High (delayed granulomas)
PMMA	No	Very high
Silicone	No	Very high

Severe complications included intra-arterial injections resulting in necrosis and visual symptoms (e.g., blurred vision and blindness) [5]. Forehead, glabellar, nose, and nasal tip injections were significantly associated with intra-arterial complications. Filler material enters the lumen of the dorsal nasal artery, supratrochlear or angular artery, and migrates into the ophthalmic artery, traveling retrograde through a branch, resulting in necrosis and visual symptoms. Although exceedingly rare (1:10 million - 1:1 million), the consequences are catastrophic [6, 7]. Injections with Radiesse (calcium hydroxyapatite) were associated considerably with intra-arterial injections resulting in necrosis and visual symptoms, initially thought to be related to particle size. Recent work has shown that smaller particles could also block the terminal arterioles, resulting in tissue necrosis, even if the anatomic area is generally well perfused [8]. In essence, it might be the difficult filler flow through the needle(s) that caused inadvertent blockage of vessels in the target areas. Similarly, Sculptra caused nodule formation could be due to the clumps formed with the old FDA-approved reconstitution protocol. Since the adoption of the new reconstitution protocol, Sculptra related nodule formation has been much reduced. Anatomic distribution revealed: cheek 32%, nasolabial folds 14.6%, and lips 17.9% [5]. Commonly reported adverse events included swelling in 60.1%, nodules 33.7%, and pain 22.6% [5]. Most filler-related adverse effects were not always specific to the type of filler, and both short- and long-lasting fillers come with the risk of lifelong complications, e.g., excessive volume augmentation would lead to skin necrosis for most fillers regardless of type; similarly, poor patient-selection, injection technique, unclear indications, wrong selection of placement sites, injection needle, or filler, or post-injection infection caused by contaminated ice or water could cause complications regardless of specific material.

The Executive Summary from 2025 provides regulatory guidelines and an additional communication from October 2025 reiterates that (a). The FDA has not approved injectable silicone or any injectable fillers for body contouring or enhancement, which can lead to serious injury, including long-term pain, infection, permanent scarring or disfigurement, and even death; and (b). The FDA also indicates that needle-free devices for the injection of dermal fillers are not approved. The high pressure they require do not provide enough control over where filler will be placed, potentially causing serious injuries and, in some cases, permanent harm to the skin, lips, or eyes [1, 9].

Similarly, injection of filler into other named vessels, such as the angular artery or supratrochlear artery, can also cause vascular compromise. A thorough knowledge of arterial anatomy is necessary before injection, since it is well known that occasionally even the routinely used, safe practice of needle aspiration is not effective and may not flash blood [10]. Larger particle size (calcium hydroxyapatite) could block vessels, much larger and more proximal vessels, causing cerebral infarction. Routinely recommended preventive measures, such as blanching, are unreliable in some DFs due to their higher viscosity and dense opacity [11]. Additionally, larger needle sizes are associated with a higher risk of vascular occlusion due to more likely retrograde flow [12]. This complication is an emergency: Permanent vision loss is imminent, and quick intervention is crucial. Recognition and management of vascular compromise are paramount. Prompt injection of high-dose hyaluronidase is required in HA filler-associated occlusion. Ultrasound guidance may assist in both injection and emergency intervention. Despite all measures, the prognosis of vision recovery is still extremely poor.

Intra-arterial injection with subsequent necrosis occurs in 0.1–0.3% of fillers, more commonly in the lip, forehead, and nasolabial fold. Incidence can increase with poor injection technique or inappropriate filler-choice, especially in areas where critical blood vessels are located. The rich vascular cascade of the face further exacerbates the effects of inadvertent intravascular injection when the filler travels to distant sites [13]. Again, larger-particle fillers, including calcium hydroxyapatite, are significantly more likely to cause skin necrosis. Occasionally, tissue necrosis can result from a larger volume of filler injected adjacent to the vasculature, causing undue pressure compressing venous outflow [14]. Blanching extending beyond the immediate injection area, followed by skin duskiness, can be an early signal of vascular intrusion [15]. Immediate cessation of the filler injection is necessary, and in extreme cases, surgical excision of non-viable tissue and secondary reconstruction with a flap of skin grafting is indicated. Sterile technique is mandatory. Preprocedural skin disinfection with chlorhexidine or alcohol reduces infection risk. Nodules and granulomas require tailored therapy, such as corticosteroids, antibiotics, or intralesional hyaluronidase for HA fillers.

Tissue atrophy occurs in <1% patients, especially with high-volume fillers or when used in areas with thinner skin and with repetitive treatments. Over time, the filler disrupts tissue architecture. Local injection of platelet-rich plasma (PRP) or stem cells can stimulate tissue regeneration and improve tissue thickness. Overall, this condition is difficult to treat, and preventive measures, including careful selection of the treatment site and filler material, are critical.

Excessive use of fillers, leading to a distorted and unnatural appearance, aka facial overfilled syndrome (FOS), is seen in anatomic sites where these deformities are common include: lips, nasal dorsum, pretarsal roll of the eyelid, nasolabial fold, malar prominence, and forehead [16, 17]. Common manifestations include “flower horn” foreheads, “sunset” eyes, “chipmunk” cheeks, “witch” chins, and “pillow” faces. Extreme augmentation using poor injection techniques and a disregard for anatomy limits tissue integrity and disrupts the aging process, and the consequences of high-pressure filling and filler migration. Prevention involves a customized approach to patients, their expectations, and careful site-selection. Facial movements, tissue thickness, bony landmarks, and most importantly, limits of filler amounts are important considerations. The etiology of FOS is multifactorial: Large amounts of filler material result in clumping facilitated by migration to an undesired location caused by facial movements and muscle contraction. The areas swell up due to water retention (most fillers are hydrophilic)—the larger tissue volume causing significant distortion of the treated areas. Initial treatment could include hyaluronidase and even surgical excision, and volume reduction of the affected areas may be indicated.

Autoimmune/inflammatory syndrome induced by adjuvants (ASIA) syndrome was first defined in 2011, and describes abnormal innate and adaptive immune responses to adjuvants, including HA fillers [18]. Post-injection inflammation and tissue damage are followed by an autoimmune reaction mediated by dysregulated T and B-lymphocytes. Specific HLA antigens are implicated [19]. Clinically, muscle weakness, joint pain, and fatigue are common symptoms that can manifest several years after filler injection. Due to this and a lack of proximal causality, the diagnosis is often delayed.

*Edema vs. hematoma*: DFs commonly cause tissue edema, especially hydrophilic agents like HA. Differentiating edema from a hematoma is difficult [20]. Clinically, a hematoma presents with bruising in a specific area of the filler site, as opposed to a generalized swelling resulting from water retention. Close post-injection monitoring is crucial to diagnose both and tell them apart.

*Dermatitis*: Occasionally, dermal fillers cause severe itching, redness, blistering, and other immunological tissue reactions. In some patients, dermatitis can persist for a long duration. They represent reactions to specific constituents of DFs such as carboxymethylcellulose (CMC) in calcium hydroxyapatite, Facestem, Elansé, and Rykoll [21].

*Persistent erythema*: Some patients develop a delayed, diffuse erythema weeks after injection, which can persist for a prolonged duration. Clinically, round or ovoid erythematous patches appear at the injection site. It is due to compression of either the superficial skin or where tissues are compact and adherent to fascia/bone, with little space for expansion. Pressure caused by the extra volume from the filler impedes venous outflow, affects capillary pressures, causing erythema, and could also result in permanent changes from compensatory neovascularization [22]. Local application of steroids and laser are useful in many cases. When this occurs early, diffuse erythema can mask more serious tissue necrosis or even infection. Clinically, early signs of tissue necrosis present with a purple/red reticular patch starting within 3-4 days post-injection [23].

*Granuloma/Nodule formation*: Granulomas occur in approximately 1 to 2% of patients, particularly with polymethylmethacrylate (PMMA) dermal fillers, such as Sculptra, which are permanent or semi-permanent fillers that generate an inflammatory reaction around the filler material itself. Collagen injections have a risk of approximately 6% to produce granulomatous reactions that initially present as a nodule, then grow and become permanent. These can increase the chances of granuloma formation in the future. Differentiation of granulomas from other nodules requires radiolabeled leukocyte scintigraphy [15]. With granulomas, although the risk of developing autoimmune diseases and acute hypersensitivity reactions is very rare, persistent edema, sarcoid-like reactions, and panniculitis have been observed (2, 23). Initial treatment is prevention of nodule formation, instructing the patient to massage the area, 5 times a day for 5 minutes for 5 days. Surgical excision is needed for granulomas that are unresponsive, especially because more granulomas could develop in response to the foreign material.

*Tyndall effect*: Superficial injection of hyaluronic acid fillers can cause light scattering of shorter wavelengths, leading to a bluish discoloration known as the Tyndall effect [24]. This is more common in areas where the skin is thinner, like the tear trough, perioral area, or smoker’s lines. Prevention requires deeper interventions in these areas [25]. Similarly, since more HA-particles scatter more light, using smaller quantities of filler is essential. Additionally, manipulating the filler area by massage can help distribute it more evenly, especially if initiated as soon as the bluish tinge is observed [26]. Surgical drainage or aspiration of the filler is recommended to treat this, since the filler tends to pool and encapsulate itself.

*Post-inflammatory hyperpigmentation (PIH)*: Patients with Fitzpatrick skin types IV or VI are prone to developing PIH after filler injections. Largely caused by inflammation and melanocytic recruitment to the injection sites, PIH can be prevented by minimizing the number of injection points using the smaller needles and reducing entry sites. Skin lightening via hydroquinone or laser therapies is effective [21].

*Migration*: Filler migration occurs in up to 1% of cases and causes a suboptimal outcome, like an uneven contour, with a lumpy appearance that is permanent when left untreated. Improper filler placement, muscle movement from facial expressions, and external pressure can facilitate migration. Gently massaging the filler can, in some cases, reposition the filler material when diagnosed early. Combining this with hyaluronidase for HA fillers is effective, and if unresponsive, surgical excision and removal are needed. Patients with a severe deformity caused by migration of a large volume of material need surgical excision and may require reconstruction [27].

*Infection*: Resulting from contaminated materials, injection needles, diluents including water/saline, or preexisting skin infections, filler–site infections are largely preventable. Manipulation close to an injection site may result in infection even years after the placement of the filler [28]. Empiric use of preventative post-procedure antibiotics is frowned upon but routinely practiced. Once diagnosed, a thorough assessment of infectious sources and abscess formation is crucial. Additional use of macrolides is also effective. Some infections, especially in perioral areas, could arise from Herpesvirus, requiring antiviral treatment. Key cross-article conclusions consistent across systematic reviews and meta-analyses include: Permanent complications are overwhelmingly vascular in origin**;** permanent fillers disproportionately contribute to late complications**;** anatomic site matters more than filler volume**;** blindness remains irreversible in nearly all reported cases**;** HA fillers dominate severe complication reports due to volume of use, not intrinsic risk. It must be emphasized that since HA fillers are dissolvable over time, they are preferable to non-dissolving permanent options, including PMMA, for a variety of reasons: Unfavorable results can be treated with hyaluronidase to hasten dissolution, and complications can be reversed more easily than PMMA, which requires physical removal.

*Medicolegal implications*: The expanded pool of medical and non-medical providers, variable settings from clinics, med-spas, and even medical tourism increase the risk of complications and their medicolegal implications. Informed consent, a critical step in any procedure, can often be inadequate in these settings [29, 30] (Fig. 6). Median awards in favor of the plaintiff or settlement ranged from $262,000- $600,000—the most common factors cited include: practitioner’s lack of expertise, lack of informed consent, and delayed diagnosis leading to a poor outcome [5, 31].

## Discussion

**Fig. 7:**
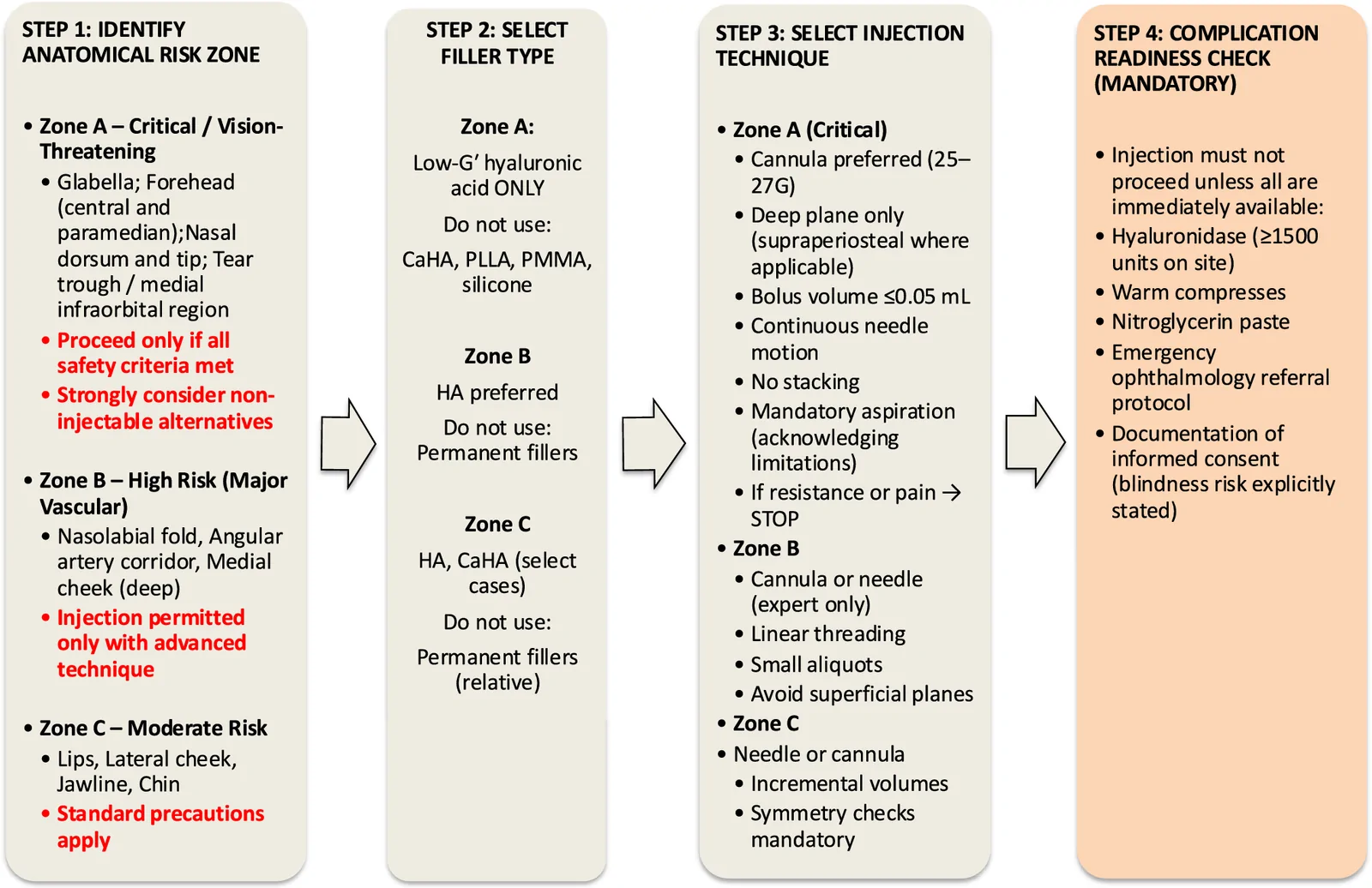
Clinical decision tree that will help avoid complications

This study provides a multifaceted examination of permanent complications of dermal fillers, including potential etiologies, costs of complications, medicolegal issues, effective treatments, and prevention. Based on these findings, we have created a framework of safe use of dermal fillers. Based on the recommendations in the literature, we have created a clinical decision tree (CDT) (Fig. 7). The CDT takes into account anatomic considerations, filler selection, injection techniques, and preparedness for complications.

*Anatomic considerations*: A thorough understanding of facial anatomy and danger zones, and knowledge of regional anatomy and high-risk vascular zones are essential. In the glabella and forehead, the branches of the supratrochlear and supraorbital arteries can create a risk for vascular occlusion and potential blindness if filler is inadvertently injected intravascularly. The nose and nasolabial fold are rich in arterial networks, including the angular artery, posing a heightened vascular risk. In both these areas, small incremental injections and aspiration are recommended. Similarly, in the perioral regions (lips and chin), the mental and inferior labial arteries reside in deeper planes. Hence, injections should be superficial to avoid vessel compromise.

*Injection technique*: The prudent selection of a needle or a cannula varies by anatomic location: Cannulas are recommended in high-risk vascular areas (e.g., glabella, nose) since their blunted tips reduce the risk of vessel penetration. Needles allow precise placement in superficial regions like lips or tear troughs but require careful aspiration and slow linear threading to avoid intravascular injection. Proper aspiration before injection remains a debated but widely endorsed safety step, especially in high-risk anatomic planes. In general, injections must be slow, with minimal force and small volume per pass, as this decreases inadvertent mechanical disruption of vessels. Sterile technique is mandatory. Preprocedural skin disinfection with chlorhexidine or alcohol reduces infection risk. Nodules and granulomas require tailored therapy, such as corticosteroids, antibiotics, or intralesional hyaluronidase for HA fillers.

*Filler selection*: Selection of the right filler for the right indication is the first and foremost consideration. Usually, the type of filler is based on anatomic location and product properties. Filler selection should account for rheological properties (viscosity, elasticity) and reversibility. Hyaluronic Acid (HA): preferred for most facial areas due to reversibility with hyaluronidase in case of complications. Calcium Hydroxyapatite or PLLA: firmer fillers for deep volumization (e.g., cheeks, jawline) but with less reversibility. Permanent fillers (e.g., PMMA, silicone): associated with lifelong complications and should be used with extreme caution or avoided altogether. Taken together, these factors are summarized in Table 5. Best practice injection techniques emphasize constant movement, perfect layer selection (deep vs. superficial), smaller injection volume boluses, selection of known avascular spaces for filler placement, and selecting the right cannula sizes.

**Table 5:** Safe selection and injection techniques based on anatomic location

Location	Preferred fillers	Considerations
Cheeks/cheekbones	HA high G′ or CaHA	Deep supraperiosteal placement; avoid superficial lumps
Tear trough/under eye	Low viscosity HA	Superficial placement; minimal product to avoid Tyndall effect
Lips	HA tailored for mobility	Small aliquots; symmetrical injection to prevent asymmetry
Nose	HA via cannula	Slow injections; preferential to avoid vascular structures

*Prevention and management of complications rely on awareness, early diagnosis, and prompt treatment*. Recognition and management of vascular compromise are paramount. Prompt injection of high-dose hyaluronidase is required in HA filler-associated occlusion. Ultrasound guidance may assist in both injection and emergency intervention.

Preoperative consultation and screening of medical history, risk factors, and managing patient expectations are essential for all patients. Despite the currently popular trends of overfilled faces and lips, providers need to have an open discussion with patients about the potential hazards. Providing examples is instructive and facilitates informed consent principles. Education on post-filler care practices and vigilance is important, especially with the rise of medical tourism, wherein patients with complications from unnamed and sometimes undisclosed fillers are seen.

Despite all these precautions, adverse effects may occur even in the best hands. In these situations, prompt diagnosis and treatment are time-sensitive.

Critics argue that stronger regulatory frameworks are needed, but in practice, they are difficult to enforce. Patient safety is the main responsibility of the provider, whether they are plastic, dermatologic, or cosmetic surgeons, anesthesiologists, OB/gyn, family practitioners, nurse practitioners, or physician assistants. Laws allow only licensed professionals to administer fillers, and the ultimate responsibility for best practice guidelines lies with them. Development of standardized protocols for dosage, storage quality standards, and injections, and mandatory adverse effect reporting is crucial to improving patient safety, guiding treatments, and empowering patients to make informed decisions.

*Study limitations*: This review offers a comprehensive overview of dermal filler side effects and complications, utilizing a wide range of studies from reputable databases. Categorizing side effects into early and delayed types of aids in timely identification and intervention. Exclusion of non-English studies, potential heterogeneity among the included studies, a focus on short-term outcomes with limited long-term data, and possible publication bias favoring positive outcomes.

## Conclusions

Prevention and management of both temporary and permanent adverse effects require proper selection, technique, and prompt recognition and management. Provider training, patient education, a robust reporting mechanism, and regulatory vigilance could further enhance patient safety and yield better outcomes.

## Supplementary Information

Below is the link to the electronic supplementary material.Supplementary file1 (DOCX 130 KB)
