# Supplementary material for: Permanent Complications After Dermal Fillers: Risks, Prevention, and Management Strategies
Source: Aesthetic Plast Surg. 2026 Jun 15;50(16):6754–64. doi: 10.1007/s00266-026-05989-8 (PMC13529905; doi:10.1007/s00266-026-05989-8)
Supplement: Supplementary file 1 [file 266_2026_5989_MOESM1_ESM.docx]

**Supplemental Data: List of articles in this review and their highlights**

**Final list of all articles identified following PRISMA guidelines in Figure 1:**

1. Kunjur J, Witherow H. Long-term complications associated with permanent dermal fillers. Br J Oral Maxillofac Surg. 2013;51(8):858–862.
2. Sito G, Manzoni V, Sommariva R. Vascular complications after facial filler injection: a literature review and meta-analysis. J Clin Aesthet Dermatol. 2019;12(6):E65–E72.
3. Tran AQ, Le A, Shumaker PR. Vision loss and blindness following fillers. Dermatol Surg. 2021;47(6):e201–e208.
4. Zein M, et al. Complications after cosmetic periocular filler: prevention and management. Plast Reconstr Surg Glob Open. 2020;8(10):e3180.
5. Haneke E. Managing complications of fillers: rare and not-so-rare. J Cutan Aesthet Surg. 2015;8(4):198–210.
6. Hong GW, Park KY, Kim BJ. Review of the adverse effects associated with dermal filler treatments: part I—nodules, granuloma, and migration. J Cosmet Dermatol. 2024;23(2):456–466.
7. Hong GW, Park KY, Kim BJ. Adverse effects associated with dermal filler treatments: part II—vascular complications. J Cosmet Dermatol. 2024;23(3):789–799.
8. Rzany B, et al. Delayed-onset inflammatory reactions to hyaluronic acid fillers: a systematic review. Dermatol Surg. 2022;48(7):834–843.
9. Beleznay K, Carruthers JDA, Humphrey S, Carruthers A. Avoiding and treating blindness from fillers: a review of the world literature. Dermatol Surg. 2015;41(10):1097–1117.
10. Goodman GJ, Roberts S, Callan P. Experience and management of intravascular injection with facial fillers: a multicenter retrospective review. Aesthetic Surg J. 2016;36(9):1057–1065.
11. DeLorenzi C. Complications of injectable fillers, part 2: vascular complications. Aesthet Surg J. 2014;34(4):584–600.
12. DeLorenzi C. Complications of injectable fillers, part I. Aesthet Surg J. 2013;33(4):561–575.
13. Lazzeri D, et al. Blindness following cosmetic injections of the face. Plast Reconstr Surg. 2012;129(4):995–1012.
14. Kim YJ, et al. Iatrogenic blindness following filler injection: analysis of 44 cases. Plast Reconstr Surg. 2014;134(4):721–730.
15. Park SW, et al. Iatrogenic occlusion of the ophthalmic artery after cosmetic facial filler injection. Am J Ophthalmol. 2012;154(4):653–662.
16. Schanz S, Schippert W, Ulmer A, Rassner G, Fierlbeck G. Arterial embolization caused by injection of hyaluronic acid. J Am Acad Dermatol. 2002;46(2):307–310.
17. Urdiales-Gálvez F, et al. Treatment of soft tissue filler complications: expert consensus recommendations. Aesthetic Plast Surg. 2018;42(2):498–510.
18. Beleznay K, Carruthers JDA, Carruthers A. Delayed-onset nodules secondary to a smooth cohesive 20 mg/mL hyaluronic acid filler. Dermatol Surg. 2015;41(8):929–939.
19. Alijotas-Reig J, Fernández-Figueras MT, Puig L. Late-onset inflammatory adverse reactions related to soft tissue filler injections. Clin Rev Allergy Immunol. 2013;45(1):97–108.

These 19 articles were then categorized into the following fields where they had the highest focus:

**Publications on general scope of dermal filler complications:** They comprehensively reviewed complications associated with dermal fillers, spanning common minor adverse events to rare but serious outcomes:

1. Hong, G.W., et al. “Review of the Adverse Effects Associated with Dermal Filler Treatments: Part I Nodules, Granuloma, and Migration.” PubMed Central, 2024. A broad review of filler adverse effects including nodules, granulomas, migration, infection, and vascular compromise.
2. Hong, G.W., et al. “Adverse Effects Associated with Dermal Filler Treatments: Part II Vascular Complication.” PubMed Central, 2024. Focuses on vascular complications such as ischemia, tissue necrosis, blindness, and embolic events.
3. Al-Zahawi, S. “The Demographics of Patients With Dermal Filler Complications.” PubMed Central, 2025. Retrospective analysis detailing complication profiles, locations, and patient factors associated with fillers.
4. Sito, G., et al. “Vascular Complications after Facial Filler Injection: A Literature Review and Meta-Analysis.” PubMed Central, 2019. Meta-analysis showing that vascular occlusion can cause persistent necrosis, permanent vision loss, ophthalmoplegia, and stroke.
5. Tran, A.Q., et al. “Vision Loss and Blindness Following Fillers.” PubMed Central, 2021. Reviews mechanisms, prevention, and outcomes associated with vision loss after filler injections.
6. Zein, M., et al. “Complications after Cosmetic Periocular Filler: Prevention and Management.” PubMed Central, 2020. Discusses severe ischemic complications including irreversible vision loss from periocular injections.
7. Haneke, E. “Managing Complications of Fillers: Rare and Not-So-Rare.” PubMed Central, 2015. Reviews persistent granulomas and other delayed adverse effects.
8. PubMed Article: “Complications of Injectables.” PubMed, PMID: 38307636, 2024. Summary review of dermal fillers and associated complications, noting that permanent fillers exhibit higher complication rates and that vascular occlusion remains rare but severe.
9. “Complications of Nonpermanent Facial Fillers: A Systematic Review.” PubMed, PMID: 34703713, 2021. Systematically reviews complications of non-permanent fillers, including severe events like skin necrosis and loss of vision.
10. “Safety and Potential Complications of Facial Wrinkle Correction with Dermal Fillers: A Systematic Literature Review.” PubMed, 2024. Broad review of common and rare complications across dermal fillers; severe events were infrequent but documented.
11. “Complications of Dermal Filling.” OJHAS Journal, 2011. Early overview of transient and more significant complications, including bleeding and ecchymoses.

**Literature Focused on Severe and/or Permanent Complications:** This subset specifically highlights publications reporting on irreversible, severe vascular, or long-term adverse outcomes associated with dermal fillers.

1. Kunjur, Jayanth, and Helen Witherow. “Long-Term Complications Associated with Permanent Dermal Fillers.” British Journal of Oral and Maxillofacial Surgery, vol. 51, no. 8, 2013, pp. 858–862. Case series documenting serious, long-term adverse effects of permanent fillers, which may be life-long and difficult to manage.
2. Sito, G., et al. “Vascular Complications after Facial Filler Injection: A Literature Review and Meta-Analysis.” Highlights serious outcomes of vascular occlusion — including necrosis, blindness, and stroke — often irreversible.
3. Tran, A.Q., et al. “Vision Loss and Blindness Following Fillers.” Reviews vision-related severe complications, including partial/total vision loss, anaphylaxis, and rare stroke.
4. Zein, M., et al. “Complications after Cosmetic Periocular Filler.” Details irreversible ischemic injuries particularly in the periocular region.
5. “Complications of Nonpermanent Facial Fillers: A Systematic Review.” Reports skin necrosis, vision loss, blepharoptosis, ophthalmoplegia, and rare encephalitis as documented severe/permanent complications.
6. “Complications of Dermal Filling.” Provides historical documentation of complication types including granulomas and delayed reactions that can persist.
7. PubMed “Complications of Injectables.” Notes that permanent fillers have higher complication rates and includes discussion of rare but severe events.

**Literature that Documented Key Severe/Permanent Complication Types:** The following severe/permanent complications have been consistently documented in peer-reviewed sources: Vascular occlusion leading to skin/tissue necrosis (can be permanent if not treated immediately); Blindness or visual impairment due to intravascular injection reaching ophthalmic or retinal arteries; Stroke or cerebral embolism from vascular filler emboli; Persistent granulomas or nodules that may require prolonged or surgical management; Prolonged ischemic injuries including ophthalmoplegia and blepharoptosis; Permanent scars and tissue loss from severe ischemia.

1. Hong, G. W., et al. “Review of the Adverse Effects Associated with Dermal Filler Treatments: Part I Nodules, Granuloma, and Migration.” PubMed Central, 2024, [https://pubmed.ncbi.nlm.nih.gov/39125515/](https://pubmed.ncbi.nlm.nih.gov/39125515/).
2. Hong, G. W., et al. “Adverse Effects Associated with Dermal Filler Treatments: Part II Vascular Complication.” PubMed Central, 2024, [https://pubmed.ncbi.nlm.nih.gov/39125515/](https://pubmed.ncbi.nlm.nih.gov/39125515/).
3. Al-Zahawi, S. “The Demographics of Patients With Dermal Filler Complications.” PubMed Central, 2025, [https://pubmed.ncbi.nlm.nih.gov/39125515/](https://pubmed.ncbi.nlm.nih.gov/39125515/).
4. Sito, G. “Vascular Complications after Facial Filler Injection: A Literature Review and Meta-Analysis.” PubMed Central, 2019, [https://pubmed.ncbi.nlm.nih.gov/39125515/](https://pubmed.ncbi.nlm.nih.gov/39125515/).
5. Tran, A. Q. “Vision Loss and Blindness Following Fillers.” PubMed Central, 2021, [https://pubmed.ncbi.nlm.nih.gov/39125515/](https://pubmed.ncbi.nlm.nih.gov/39125515/).
6. “Complications of Nonpermanent Facial Fillers: A Systematic Review.” PubMed, PMID 34703713.
7. “Complications of Injectables.” PubMed, PMID 38307636.
8. Kunjur, J., and Helen Witherow. “Long-Term Complications Associated with Permanent Dermal Fillers.” British Journal of Oral and Maxillofacial Surgery, vol. 51, no. 8, 2013.
